# Expression analysis of mammaglobin A (*SCGB2A2*) and lipophilin B (*SCGB1D2*) in more than 300 human tumors and matching normal tissues reveals their co-expression in gynecologic malignancies

**DOI:** 10.1186/1471-2407-6-88

**Published:** 2006-04-09

**Authors:** Menelaos Zafrakas, Beate Petschke, Andreas Donner, Florian Fritzsche, Glen Kristiansen, Ruth Knüchel, Edgar Dahl

**Affiliations:** 1 Institute of Pathology, University Hospital Aachen, RWTH Aachen, Pauwelsstrasse 30, 52074 Aachen, Germany; 2 Department of Obstetrics and Gynecology, Charité University Hospital, Campus Virchow, Augustenburger Platz 1, 13353 Berlin, Germany; 3 Institute of Pathology, Charité, Universitätsmedizin Berlin, Schumannstr. 20/21, 10117 Berlin, Germany

## Abstract

**Background:**

Mammaglobin A (*SCGB2A2*) and lipophilin B (*SCGB1D2*), two members of the secretoglobin superfamily, are known to be co-expressed in breast cancer, where their proteins form a covalent complex. Based on the relatively high tissue-specific expression pattern, it has been proposed that the mammaglobin A protein and/or its complex with lipophilin B could be used in breast cancer diagnosis and treatment. In view of these clinical implications, the aim of the present study was to analyze the expression of both genes in a large panel of human solid tumors (n = 309), corresponding normal tissues (n = 309) and cell lines (n = 11), in order to evaluate their tissue specific expression and co-expression pattern.

**Methods:**

For gene and protein expression analyses, northern blot, dot blot hybridization of matched tumor/normal arrays (cancer profiling arrays), quantitative RT-PCR, non-radioisotopic RNA *in situ *hybridization and immunohistochemistry were used.

**Results:**

Cancer profiling array data demonstrated that mammaglobin A and lipophilin B expression is not restricted to normal and malignant breast tissue. Both genes were abundantly expressed in tumors of the female genital tract, i.e. endometrial, ovarian and cervical cancer. In these four tissues the expression pattern of mammaglobin A and lipophilin B was highly concordant, with both genes being down-, up- or not regulated in the same tissue samples. In breast tissue, mammaglobin A expression was down-regulated in 49% and up-regulated in 12% of breast tumor specimens compared with matching normal tissues, while lipophilin B was down-regulated in 59% and up-regulated in 3% of cases. In endometrial tissue, expression of mammaglobin A and lipophilin B was clearly up-regulated in tumors (47% and 49% respectively). Both genes exhibited down-regulation in 22% of endometrial tumors. The only exceptions to this concordance of mammaglobin A/lipophilin B expression were normal and malignant tissues of prostate and kidney, where only lipophilin B was abundantly expressed and mammaglobin A was entirely absent. RNA *in situ *hybridization and immunohistochemistry confirmed expression of mammaglobin A on a cellular level in endometrial and cervical cancer and their corresponding normal tissues.

**Conclusion:**

Altogether, these data suggest that expression of mammaglobin A and lipophilin B might be controlled in different tissues by the same regulatory transcriptional mechanisms. Diagnostic assays based on mammaglobin A expression and/or the mammaglobin A/lipophilin B complex appear to be less specific for breast cancer, but with a broader spectrum of potential applications, which includes gynecologic malignancies.

## Background

Mammaglobin A (secretoglobin, family 2A, member 2 – *SCGB2A2*) and lipophilin B (secretoglobin, family 1D, member 2 – *SCGB1D2*) are members of the secretoglobin superfamily, a group of small, secretory, rarely glycosylated, dimeric proteins with unclear physiologic functions, mainly expressed in mucosal tissues [1,2]. The rabbit uteroglobin is the founder member of this family of mammalian proteins [1], which has expanded to more than 25 members in recent years, currently including nine human secretoglobins. Mammaglobin A, lipophilin B, and most of the human secretoglobins are localized on chromosome 11q13, where they form a dense cluster [1].

The mammaglobin A gene (*SCGB2A2*) encodes a 93-amino acid protein with a predicted molecular mass of 10.5 kDa [3,4]. In breast tissue it exists in two main forms with approximate molecular masses of 18 and 25 kDa, due to posttranslational modifications [5]. Mammaglobin A is considered to be a highly specific breast tissue marker; initially it was found to be overexpressed in breast cancer, and its expression was restricted to normal and malignant breast tissue [3,4]. No gene amplification or gene rearrangement was detected in tumors overexpressing mammaglobin A, suggesting changes in transcriptional regulation as the cause of overexpression [4]. In contrast to other members of the secretoglobin family [6], its expression does not appear to be influenced by steroid hormones [4,7].

Due to its tissue specificity, mammaglobin A has drawn much attention with more than 70 relevant publications in the last five years. More than 30 studies have evaluated its role in detection of minimal residual disease in breast cancer patients, while others investigated its role as a diagnostic and prognostic marker, and its potential use as a therapeutic target (see Ref. 8 for review). Recently however, some studies have shown that it is also expressed in tissues other than the breast [7,9-14]. In breast cancer mammaglobin A is overexpressed in a high proportion of primary tumors [7,14-17], and it is associated with estrogen receptor positive tumors, a less aggressive tumor phenotype [7,14,15,17], and relapse-free survival [7].

Lipophilin B (*SCGB1D2*) has not been studied as extensively as mammaglobin A. The secreted lipophilins A, B, and C should not be confused with the family of lipophilins described as hydrophobic integral membrane proteins in myelin [1]. Lipophilin B is expressed in a high proportion of breast carcinomas [14,18], it is more frequently expressed in estrogen receptor positive tumors [14], but it shows a lower degree of tissue-specificity [18]. Recently, two studies independently showed that in breast cancer the mammaglobin A and lipophilin B proteins form a covalent complex, and that the two proteins are bonded in a head-to-tail orientation [19,20]. Moreover, the expression levels of mammaglobin A in breast tumors were significantly correlated with those of lipophilin B [14,19,20].

The association between mammaglobin A and lipophilin B in breast cancer, the controversy about tissue-specificity of mammaglobin A, and the clinical implications by the use of both genes in cancer early detection, diagnosis, and treatment gave us the impetus to systematically analyse their expression in a large panel of normal and malignant human tissues and cell lines. We report herein that mammaglobin A expression and its co-expression with lipophilin B are not restricted to breast cancer, and that their applications in cancer diagnosis and treatment could also include malignancies of the female genital tract.

## Methods

### Tissue specimens and cell lines

Formalin-fixed paraffin-embedded tissue from cervical, endometrial and breast cancer and corresponding normal tissue specimens were obtained from patients treated at the Gynecology Departments of the Charité Berlin and the University Hospital of Aachen, with institutional review board approval. Cell lines were obtained from ATCC and cultured as described in the ATCC cell biology catalogue (LGC Promochem, Teddington, England). The following 11 cell lines were analyzed by RT-PCR: HaCat (human keratinocytes), MCF-10A (breast tissue, fibrocystic disease), T47D (breast cancer), ZR75.1 (breast cancer), MDA-MB 468 (breast cancer), MDA-MB 231 (breast cancer), PC3 (prostate cancer), LnCaP (prostate cancer), DU 145 (prostate cancer), MaTu (breast cancer), and A375 (malignant melanoma).

### Multiple tissue northern blot in malignant and normal breast tissue

Mammaglobin A and lipophilin B expression was analyzed on a Clontech (Heidelberg, Germany) multiple tissue northern blot containing four pairs of invasive ductal carcinoma and matched normal tissue from four female patients (51, 36, 47, and 45 years old). Hybridization was performed as described in the following section for the tumor/normal cDNA arrays.

### Expression analysis using tumor/normal cDNA arrays

Mammaglobin A and lipophilin B expression were each analyzed using two different nylon filter arrays from Clontech (Heidelberg, Germany), each containing spotted cDNAs from tumor and corresponding normal tissue of the same patient. The "Matched Tumor/Normal Expression Array" (MTNA) (Clontech, Product number 7840) consisted of 136 cDNAs, synthesized from 68 tumor and 68 matched normal tissue specimens. The "Cancer Profiling Array" (CPA) (Clontech, Product number 7841) consisted of 511 dots with 494 cDNAs synthesized from 241 primary tumor, 241 matched normal tissue, and 12 cDNAs from metastases corresponding to 12 of the tumor/normal pairs. Each cDNA pair was independently normalized based on the expression of housekeeping genes used as controls and immobilized in separate dots [22]. Data for controls and clinicopathological parameters for each specimen can be found on the provider's website [23,24].

For both the MTNA and CPA, hybridization was performed using 25 ng of a gene-specific ^32^P-labeled cDNA probe derived from Unigene cDNA clones (*SCGB2A2*: AA513640; *SCGB1D2*: AJ224172). These gene-specific cDNA fragments were radiolabelled using a Megaprime labelling kit (Amersham Biosciences, Braunschweig, Germany), hybridized overnight at 68°C using ExpressHyb Hybridization Solution (Clontech, Heidelberg, Germany), washed, and exposed to Kodak XAR-5 X-ray film with an intensifying screen (Eastman Kodak Co, Rochester, NY, USA). The tumor/normal intensity ratio was calculated using a Typhoon 9410 High Performance Imager (GE-Healthcare, Chalfont St. Giles, UK) and normalized against the background.

The specificity of the mammaglobin A and lipophilin B hybridization probes was determined by co-hybridization of nylon membranes containing different concentrations of spotted mammaglobin A and lipophilin B cDNAs in plasmid clones: 1 ng, 100 pg, 10 pg and 1 pg of cDNA from each gene were diluted in 100 ul of 15XSSC buffer, heat-denatured for 5 min by boiling and then quenched on ice. Denatured cDNAs were spotted on Hybond N+ membranes (Amersham Biosciences, Freiburg, Germany) using a vacuum manifold (Millipore, Eschborn, Germany). These membranes were treated during filter hybridization, washing and exposition exactly like the tumor/normal arrays

### Quantitative RT-PCR

Mammaglobin A and lipophilin B expression were analyzed with real-time RT-PCR in a panel of 11 cell lines (see above) and 23 normal human tissues (see Figures 5 and 6) using commercially available RNA (Clontech, Heidelberg, Germany). For each cDNA synthesis, 1μg of RNA was reverse transcribed using the Superscript II Reverse Transcription System (Invitrogen, Karlsruhe, Germany), according to the instructions of the manufacturer.

Real-time RT-PCR was performed with the Gen Amp^® ^5700 sequence detection system (PE Applied Biosystems, Weiterstadt, Germany), using intron-spanning primers and FAM (5' end)/TAMRA (3' end) – labeled specific oligonucleotides. The housekeeping gene *GAPDH *was used as reference. Primers and probes used in this study are presented in Table 1. Each PCR reaction was performed in a 25μl volume, which included 12.5μl 2XTaqMan Universal PCR-Mastermix (PE Applied Biosystems, Weiterstadt, Germany), 1 ng of cDNA template, 300 nM of forward and 900 nM of reverse primer, and the specific probe for each gene (150 nM for mammaglobin A and 100 nM for lipophilin B). Gene expression was quantified by the comparative C_T _method, normalizing C_T_-values to the housekeeping gene *GAPDH *and calculating the relative expression values of tumor and normal tissues [21].

### Non-radioisotopic RNA in situ hybridization

Non-radioisotopic RNA *in situ *hybridization in cervical and endometrial cancer and matched normal tissue was performed as previously described [25].

### Immunohistochemistry

Formalin-fixed paraffin embedded tissue was freshly cut (4μm). The sections were mounted on superfrost slides (Menzel Gläser, Braunschweig, Germany), deparaffinized with xylene and gradually hydrated. We used a monoclonal anti-mammaglobin A antibody (BioPrime, NY, USA, MAM001-05, dilution 1:100). Antigen retrieval for mammaglobin A was achieved by heat and citrate buffer using the Ventana immunostainer and all slides were stained with the BenchMark^® ^XT autostainer (Ventana, Tucson AZ, USA).

## Results

### Expression analysis using multiple tissue northern blots

**Figure 1:**
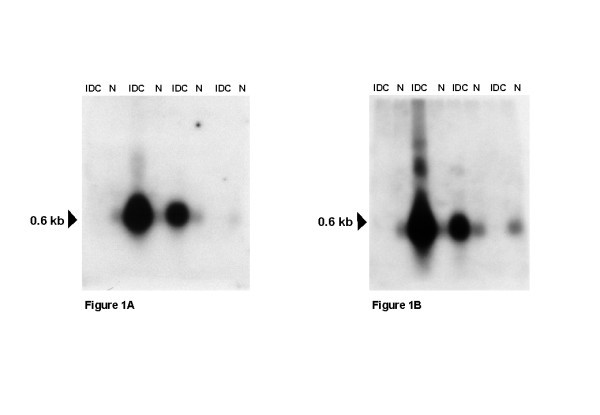
**Abundant (co)expression of mammaglobin A (*SCGB2A2*) (1A) and lipophilin B (*SCGB1D2*) (1B) in breast tumors**. Gene expression was determined using a Clontech tumor/normal northern blot (see Materials and Methods). The two genes were expressed in the same tumors, with similar hybridization signal intensities. IDC: Invasive Ductal Carcinoma; N: Normal breast tissue.

Mammaglobin A (*SCGB2A2*) and lipophilin B (*SCGB1D2*) expression was analyzed by northern blot in a panel of 4 matched breast cancer/normal breast tissue pairs (Figure 1). Transcripts of approximately 600 bp in size for both genes were expressed in the same two out of four tumor samples, with hybridization signals of similar intensity in each sample (compare Figure 1A with Figure 1B).

### Expression analysis using Cancer Profiling Arrays (CPAs)

**Figure 2:**
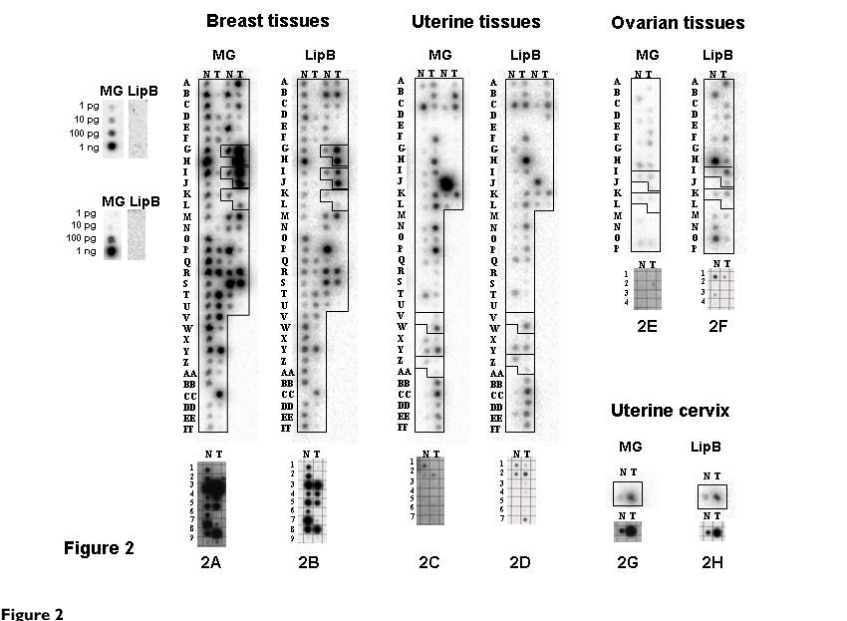
**Mammaglobin A and lipophilin B (co)expression in breast and gynecologic tumors and matched normal samples**. Expression profiles were determined using two different Clontech cancer profiling arrays for each gene (see Methods). Mammaglobin A and lipophilin B expression were each analyzed in 59 matched breast tumor/normal samples (Figures 2A and 2B), 49 matched uterine (Figures 2C and 2D), 18 matched ovarian (Figures 2E and 2F), two matched samples from the uterine cervix (Figures 2G and 2H), as well as in nine further tumor entities (data not shown). Rows N represent normal tissue, and rows T represent tumor. The outlined groups of three dots represent normal tissue (breast N: -G,  -I, -K, uterus N: -V, -Z, and ovary N: -I, -K), primary tumor (breast T:  -G, -I, -K, uterus T: -V, -Z, and ovary T: -I, -K), and metastases (breast  T: -H, -J, -L, uterus T: -W, -AA, and ovary T: -J, -L) from the same  patient. Control dots (labeled MG and LipB, respectively) were  co-hybridized in order to confirm the specificity of mammaglobin A and  lipophilin B probes (see results).

Mammaglobin A (*SCGB2A2*) and lipophilin B (*SCGB1D2*) expression were analyzed by dot blot analysis using Clontech's "Matched Tumor/Normal Array" (MTNA) and "Cancer Profiling Array I" (CPA) for each gene. The two expression arrays together contained 630 cDNAs synthesized from 309 human tumor and 309 matched normal tissue specimens, and 12 cDNAs from human metastases corresponding to 12 of the tumor/normal pairs. The specificity of the mammaglobin A and lipophilin B hybridizations on the arrays was established by co-hybridization of two dot blots, containing spotted plasmid cDNAs of either mammaglobin A or lipophilin B (see Figure 2). The radiolabelled mammaglobin A probe efficiently hybridized only to mammaglobin A cDNA (not to lipophilin B cDNA), and it was detectable up to a concentration of 1 pg. Likewise, the radiolabelled lipophilin B probe efficiently hybridized only to lipophilin B cDNA, and it was detectable up to a concentration of 10 pg. Thus, cross-hybridization was excluded between the two related genes of the secretoglobin family.

Overall, abundant expression of at least one of the two genes was detected in six of the 13 tested primary tumor entities and corresponding normal tissues. These results are summarized in Table 2. Mammaglobin A expression was abundant in malignant and normal samples from the breast (Figure 2A), uterus (Figure 2C), ovaries (Figure 2E) and uterine cervix (Figure 2G), and absent in the majority of samples from the other nine tissues (prostate, kidney, colon, rectum, small intestine, stomach, pancreas, lung, and thyroid). In the small number of metastatic samples tested its expression was heterogeneous (Figure 2). Mammaglobin A expression was also detectable in one out of 25 gastric, one out of 24 lung, one out of 34 kidney, and two out of 25 rectal tumors, but not in the corresponding normal samples (data not shown). As in the case of mammaglobin A, lipophilin B expression was abundant in malignant and normal samples from the breast (Figure 2B), uterus (Figure 2D), ovaries (Figure 2F) and uterine cervix (Figure 2H). In addition, abundant lipophilin B expression was found in matched samples from the kidney and the prostate (Table 2), while it was absent in most samples from the other seven tissues. Lipophilin B expression was also detectable in one out of 25 gastric, one out of 24 lung, and two out of 25 rectal tumors, but not in the corresponding normal samples (data not shown).

The expression pattern of mammaglobin A in malignant and normal samples from different tissues was in general highly concordant to that of lipophilin B (compare e.g. Figure 2A and 2B for breast tissue), except for kidney and prostate samples, where only lipophilin B but not mammaglobin A was expressed. The two genes exhibited an identical pattern of differential expression (i.e. both down-, up- or non-regulation) in the majority of matched pairs from the breast (78%), uterus (78%), ovaries (56%), and the uterine cervix (100%) (Table 2), without marked disparities (i.e. no cases with one gene up- and the other down-regulated) in the remaining cases. According to the Spearman rank correlation test, co-expression of mammaglobin A and lipophilin B was highly significant in breast, uterine and ovarian tissues (each p < 0.001) but failed to reach significance in cervical tissues due to the small sample size (n = 2). A very interesting finding was that mammaglobin A and lipophilin B were both up-regulated in the same one out of 25 gastric, and the same two out of 25 rectal tumor samples, in which expression of the two genes was detectable. However, this was not the case with lung tumors, in which mammaglobin A and lipophilin B were each expressed in one out of 24, but not in the same sample (data not shown). No correlation was found between the (co)expression pattern of mammaglobin A and lipophilin B in various tumors and available clinicopathological data.

### Quantitative RT-PCR

**Figure 3:**
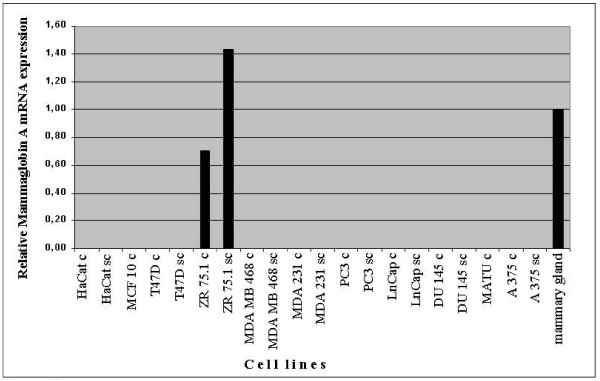
**Mammaglobin A (*SCGB2A2*) expression in human cell lines. Diagrammatic presentation of quantitative RT-PCR data**. *SCGB2A2 *mRNA was abundantly expressed in the tumorigenic breast cancer cell line ZR 75.1. *SCGB2A2 *was undetectable in four breast cancer cell lines (T47D, MDA-MB 468, MDA-MB 231, MaTu), in benign breast tissue (MCF-10A), in 3 prostate cancer cell lines (PC3, LnCaP, DU 145), in normal human keratinocytes (HaCat), and in malignant melanoma (A375). c: confluent growth; sc: subconfluent growth.

**Figure 4:**
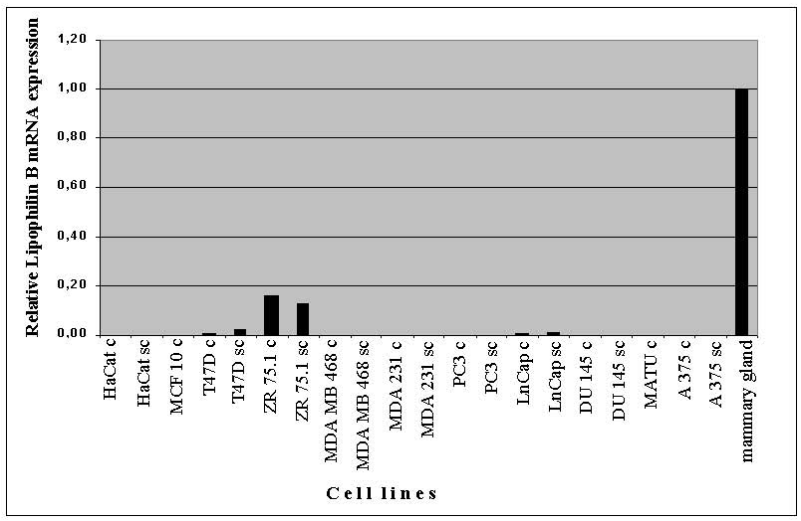
**Lipophilin B (*SCGB1D2*) expression in human cell lines. Diagrammatic presentation of quantitative RT-PCR data**. *SCGB1D2 *mRNA was expressed in two tumorigenic breast cancer cell lines (ZR 75.1 and T47D), as well as in one prostate cancer cell line (LnCaP). *SCGB1D2 *was undetectable in three breast cancer cell lines (MDA-MB 468, MDA-MB 231, MaTu), in benign breast tissue (MCF-10A), in two prostate cancer cell lines (PC3, DU 145), in normal human keratinocytes (HaCat), and in malignant melanoma (A375). c: confluent growth; sc: subconfluent growth.

Mammaglobin A (*SCGB2A2*) and lipophilin B (*SCGB1D2*) expression were analyzed with real-time RT-PCR in a panel of 11 cell lines and 23 normal human tissues using commercially available RNA (Clontech, Heidelberg, Germany). Among the cell lines tested, mammaglobin A was expressed only in the breast cancer cell line ZR-75.1, and negative in the other five breast and five non breast cell lines (Figure 3). Lipophilin B was expressed in the same cell line, as well as in the T-47D (breast cancer cell line) and LnCaP cells (prostate cancer cell line)(Figure 4). There was no major difference in expression of both genes between tumor cells grown under confluent and subconfluent conditions.

**Figure 5:**
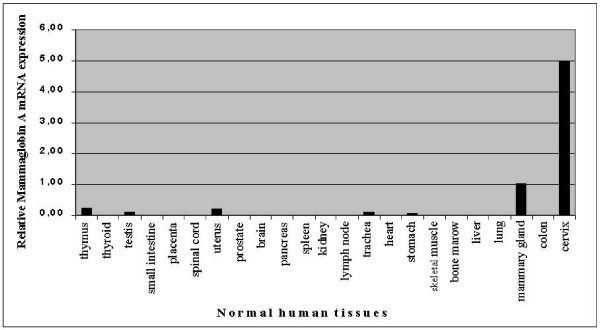
**Mammaglobin A (*SCGB2A2*) expression in human normal tissues**. Diagrammatic presentation of real-time RT-PCR data, demonstrating the level of *SCGB2A2 *mRNA expression in 23 normal human tissues. Commercially available poly A^+ ^RNA (Clontech, Heidelberg, Germany) was analyzed. Among all normal human tissues tested, normal tissue from the uterine cervix exhibited the highest level of *SCGB2A2 *expression followed by normal breast tissue.

**Figure 6:**
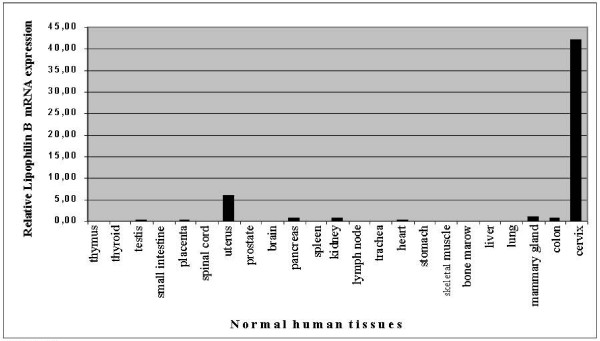
**Lipophilin B (*SCGB1D2*) expression in human normal tissues**. Diagrammatic presentation of real-time RT-PCR data, demonstrating the level of *SCGB1D2 *mRNA expression in 23 normal human tissues. Commercially available poly A^+ ^RNA (Clontech, Heidelberg, Germany) was analyzed. Among all normal human tissues tested, normal tissue from the uterine cervix exhibited the highest level of *SCGB1D2 *expression followed by normal uterine and breast tissue.

Among all normal tissues tested, mammaglobin A expression was highest in normal tissue from the uterine cervix, followed in descending order by normal breast tissue, thymus, uterus, testis, trachea, and stomach. No mammaglobin A expression was detected in the other 16 normal tissues (Figure 5). As with mammaglobin A, lipophilin B expression was highest in normal tissue from the uterine cervix. Lipophilin B was also expressed in descending order in the uterus, breast, kidney, colon, pancreas, heart, placenta, and testis. There was no detectable lipophilin B expression in the other 14 normal tissues (Figure 6).

### Non-radioisotopic RNA in situ hybridization and immunohistochemistry

**Figure 7:**
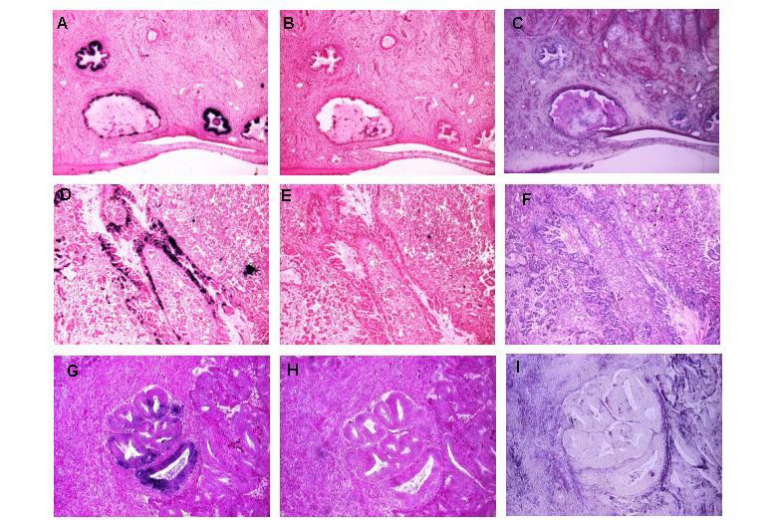
**Representative sections of mammaglobin A (*SCGB2A2*) mRNA expression as detected by non-radioactive *in situ *hybridization**. A, D, and G: Antisense *SCGB2A2 *probe; B, E, and H: Sense (control) *SCGB2A2 *probe; C, F and I: Hematoxylin/eosin stain. *SCGB2A2 *mRNA was clearly expressed in normal cervical glands (A), cervical carcinoma (D) and endometrial carcinoma sections (G).

**Figure 8:**
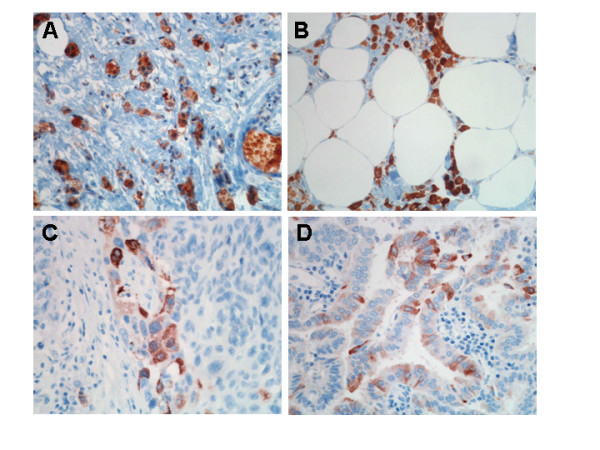
**Representative sections of mammaglobin A (SCGB2A2) protein expression as detected by immunohistochemistry**. A: Mammaglobin A expression found in invasive ductal carcinoma. B: Mammaglobin A expression in invasive lobular carcinoma. C: Mammaglobin A expression in squamous cell carcinoma of the cervix. D: Mammaglobin A expression in an endometrioid adenocarcinoma of the endometrium. Note that mammaglobin A is not expressed equally in all cells. Magnification: 400 ×.

In order to further establish the expression of mammaglobin A in gynecologic malignancies, we have analyzed its expression in tissue sections from cervical and endometrial cancer and normal tissue using non-radioisotopic RNA *in situ *hybridization. Consistently with the dot blot hybridization and quantitative RT-PCR results presented above, mammaglobin A was expressed in normal cervical glands (Figure 7A) as well as in cervical and endometrial cancer (Figure 7 D, G). Representative sections are presented in Figure 7. Furthermore we performed immunohistochemistry using a mammaglobin A-specific antibody (BioPrime, NY, USA,) on paraffin-embedded tissue from breast cancer, as well as cervical and endometrial cancer. Mammaglobin A was clearly detectable in invasive ductal (Figure 8A) and invasive lobular (Figure 8B) carcinoma of the breast. Mammaglobin A protein was also detectable in squamous cell carcinoma of the cervix (Figure 8C) and in endometrioid adenocarcinoma of the endometrium (Figure 8D).

## Discussion

In initial reports mammaglobin A appeared to be an almost ideal tissue marker, since its expression was restricted to normal and malignant breast tissue (see Ref. 8 for review). Subsequently, mammaglobin A was evaluated for detection of minimal residual disease in breast cancer patients [8,26,27], differential diagnosis of metastases and malignant pleural effusions [26-29], and as an immunotherapeutic target in *in vitro *experiments [30-33] and *in vivo *animal models [34,35]. However, in later reports, its expression was detected, rarely and/or in low levels, in various normal and malignant tissues: the normal uterine cervix [10], uterus [9-11,36], ovary [10,14,36], thymus, testis, trachea, skeletal muscle, kidney [36], skin [18], sweat glands [13], salivary glands [18,36], prostate [10], and nasal mucosa [37], and tumors of the sweat glands [13], lungs [12] and ovaries [14].

Our results confirm that mammaglobin A is expressed in various normal and malignant tissues other than the breast, and thus it is rather not an ideal breast-specific marker. Expression of mammaglobin A in normal tissues certainly limits its potential use as an immunotherapeutic target, due to concerns about autoimmune toxicity, particularly since autoimmunity is not a concern with other immunotherapeutic targets [38]. Our results, also confirm previous reports that mammaglobin A is not expressed in all breast cancer cell lines and breast tumors [3,4,7,14,16,39], and thus does not have a 100% sensitivity as a diagnostic marker. An important finding of our study was that mammaglobin A is commonly expressed in normal and malignant tissue of the female genital tract, and only rarely or at low levels in other normal and malignant tissues. It should be noted that expression in gynecologic tissues was demonstrated by four independent methods (dot blot hybridization of matched tumor/normal arrays, real time RT-PCR, non-radioisotopic RNA *in situ *hybridization and immunohistochemistry). Thus, given the limitations in specificity and sensitivity, mammaglobin A could be also used in diagnostic assays for detection of gynecologic malignancies.

The expression pattern of lipophilin B in our study, as well as in previous reports, appeared to be even less tissue specific than that of mammaglobin A, and thus its use as a diagnostic marker seems very unlikely. In the present study, lipophilin B was abundantly expressed in normal and malignant tissue from the breast, cervix, uterus, ovary, kidney and prostate. Lower or rare lipophilin B expression was found in normal colon, pancreas, heart, in gastric and rectal tumors, and as previously reported in normal testis and placenta [36] and lung tumors [12]. In previous reports, lipophilin B expression was also detected in the normal anterior pituitary and pituitary adenomas [40], in normal adrenal gland, cartilage, retina, [18], skin [19], and salivary gland [19,36].

The most important finding regarding lipophilin B expression in the present study was that it is concordant to that of mammaglobin A in most tissues tested. It has been previously reported, that mammaglobin A and lipophilin B are significantly co-expressed in breast cancer [14,19,20], and their proteins are bonded in an antiparallel manner forming a covalent complex [19,20]. In the present study we found that their co-expression is not restricted to breast tumors, but is also present in normal breast tissue, as well as normal and malignant tissue from the uterus, ovaries, and uterine cervix. On the other hand, in normal and malignant prostate and kidney tissue lipophilin B was abundantly expressed while mammaglobin A was entirely absent. Interestingly, the only gastric and two rectal tumors expressing mammaglobin A expressed lipophilin B as well, but this was not seen in lung cancer. Altogether, these data suggest that expression of the two genes, which are both localized on the same cluster on chromosome 11q13, is probably regulated by common transcriptional mechanisms. It is also reasonable to expect, that serum antibodies against lipophilin B or against its complex with mammaglobin A, as those previously detected in breast cancer patients [18], could also be found in patients with gynecologic tumors.

## Conclusion

Systematic expression analysis of a panel of solid tumors and normal tissues showed that mammaglobin A and lipophilin B are abundantly expressed in malignant and normal tissues of the breast and the female genital tract, namely the cervix, uterus, and ovary, while lower expression levels were rarely found in other tumors and normal tissues. Intriguingly, the expression pattern of the two genes was highly concordant in most tissues tested, suggesting common regulatory transcriptional mechanisms. Use of mammaglobin A and its complex with lipophilin B in breast cancer diagnosis might lead to less specific results than previously expected, but these markers could also be used in diagnosis of gynecologic cancer. The potential use of mammaglobin A as an immunotherapeutic target might be limited, due to the possibility of autoimmune toxicity.

## Abbreviations

*SCGB2A2*: secretoglobin, family 2A, member 2; *SCGB1D2*: secretoglobin, family 1D, member 2; MTNA: Matched Tumor/Normal Array; CPA: Cancer Profiling Array; RT-PCR: reverse transcription – polymerase chain reaction; GAPDH: Glyceraldehyde-3-phosphate dehydrogenase.

## Competing interests

The author(s) declare that they have no competing interests.

## Authors' contributions

MZ: participated in design of the study, data analysis, data interpretation and drafted the manuscript; BP: carried out the molecular studies, and critically revised the manuscript; AD: supported with pathological expertise in data interpretation and critically revised the manuscript; FF: established and performed the mammaglobin A immunohistochemistry analysis; GK: pathologist that analyzed the mammaglobin A immunohistochemistry study and critically revised the manuscript; RK: participated in design and coordination of the study, and critically revised the manuscript; ED conceived the study, participated in study design and coordination, molecular and data analysis, data interpretation and drafting of the manuscript.

## Pre-publication history

The pre-publication history for this paper can be accessed here:



## Figures and Tables

**Table 1:** Primers and probes used in real-time RT-PCR

**Gene**	**Primer sequence**	**Product size**
*SCGB2A2*	5'-GAACACCGACAGCAGCA-3' 5'-TCTCCAATAAGGGGCAGCC-3'	104 bp
SCGB1D2	5'-CTGAGCTCACAGCAAAAC -3' 5'-GAGCTGGGCAGAAC-3'	105 bp
GAPDH	5'-GAAGGTGAAGGTCGGAGTC-3' 5'-GAAGATGGTGATGGGATTTC-3'	226 bp

**Gene**	**TaqMan probe**	

SCGB2A2	5'-TGGTCCTCATGCTGGCGGCC-3'	
*SCGB1D2*	5'-CCATGAAGCTGTCGGTGTGTCTCCTG-3'	
GAPDH	5'-CAAGCTTCCCGTTCTCAGCC-3'	

**Table 2:** Mammaglobin A and lipophilin B (co)expression data from cDNA dot blots

		Mammaglobin A regulation *						Lipophilin B regulation *						**Concordance of expression**
**Tissue type**	**n (total)**	**up**		**down-**		**no**		**up-**		**down-**		**no**		
		**n**	**%**	**n**	**%**	**n**	**%**	**n**	**%**	**n**	**%**	**n**	**%**	

Breast	59	7	12%	29	49%	23	39%	2	3%	35	59%	22	38%	Yes (78%)
Uterus	49	23	47%	11	22%	15	31%	24	49%	11	22%	14	29%	Yes (78%)
Ovary	18	5	28%	3	17%	10	55%	4	22%	6	33%	8	45%	Yes (56%)
Cervix	2	2	100%	0	0%	0	0%	2	100%	0	0%	0	0%	Yes (100%)
Kidney	34	1	3%	0	0%	0	0%	1	3%	16	47%	17	50%	No
Prostate	7	n.e.		n.e.		n.e.		0	0%	2	29%	5	71%	No

n.e.: not expressed

* The percentage of up-regulation (or down-regulation) was defined as follows: The number of tumor/normal tissue pairs with an at least 2-fold-up-regulation (or down-regulation) in the tumor tissue divided by the total number of samples in this entity × 100. Fold changes between 0.5 and 2.0 were considered as not differentially expressed ("no regulation").
